# Early multimodal behavioral cues in autism: a micro-analytical exploration of actions, gestures and speech during naturalistic parent-child interactions

**DOI:** 10.1016/j.ijchp.2026.100664

**Published:** 2026-01-22

**Authors:** M. Mastrogiuseppe, F.I. Famà, R. Bruschetta, E. Leonardi, A. Campisi, S. Aiello, C. Carrozza, A. Ruggeri, S. Baieli, S. Campisi, L. Turriziani, G. Di Rosa, M.V. Lombardo, G. Tartarisco, O. Capirci, G. Pioggia, L. Ruta, the Nest Team

**Affiliations:** a National Research Council of Italy, Institute for Biomedical Research and Innovation (CNR-IRIB), Messina, Italy; b Centre for Autism Spectrum Disorders, Child Psychiatry Unit, Provincial Health Service of Catania, Catania, Italy; c Polyclinic Hospital “G. Martino”, Messina, Italy; d Laboratory for Autism and Neurodevelopmental Disorders, Center for Neuroscience and Cognitive Systems @UniTn, Istituto Italiano di Tecnologia, Rovereto, Italy; e National Research Council of Italy, Institute of Cognitive Sciences and Technologies, Rome, Italy

**Keywords:** Autism spectrum condition, Communication, Motor skills, Parent-child interaction, Machine learning

## Abstract

Early signs of autism often emerge through distinct developmental pathways, particularly in communication, social interaction, and play. While naturalistic parent-child interactions during free play are ideal for observing spontaneous social behaviors, few autism studies have adopted this ecological and developmental approach. To address this gap, we used a fine-grained microanalytic method to examine motor, gestural, and vocal behaviors in young children, integrating machine learning to explore how combinations of these traits distinguish early autistic neurodivergence.

We analyzed video recordings of 58 autistic and non-autistic children (aged 13–40 months) engaged in naturalistic parent-child play. A frame-by-frame micro-coding scheme was applied to capture actions, gestures, speech, and their multimodal integration.

Clear differences emerged between neurotypical (NT) and autistic (ASC) children. NT children displayed more gestures, particularly deictic and conventional-interactive, greater gesture–gaze coordination, more functional object play, and more frequent multi-word utterances. In contrast, ASC children showed fewer deictic and conventional-interactive gestures and greater use of instrumental gestures, reduced gesture–gaze coordination, a higher reliance on vocalizations rather than words, and increased object manipulation compared to functional play.

Feature selection using ANOVA F-tests identified a core set of key predictors most frequently and independently selected across folds of cross-validation: Alternate Gaze, Reaching, and Instrumental Gesture. Higher values of Alternate Gaze were associated with NT classification, while elevated frequencies of Reaching and Instrumental Gestures were linked to ASC classification. A logistic regression classifier trained on these features achieved over 85% accuracy in distinguishing the two groups.

These findings underscore the value of an ecologically valid, and developmentally informed approach to identifying early behavioral markers of autism, supporting earlier recognition and the design of more personalized, strengths-based interventions.

## Introduction

Early detection in Autism spectrum conditions (ASCs) is paramount, as it allows for the timely introduction of individualized interventions that can meaningfully support each child’s developmental pathway, fostering learning and well-being over time (Estes et al., 2015; Landa et al., 2018; Lombardo et al., 2021; Reichow et al., 2012; Warren et al., 2011). Although it is now clear that early identification is feasible with adequate diagnostic stability at much earlier ages (<18 months) (Pierce et al., 2019), the mean age of clinical diagnosis remains still delayed until 3-4 years old. Most of the studies on early autism detection are prospective studies of infants and children who are at elevated likelihood (EL) of developing autism, due to genetic loading from having an older sibling with autism (for brevity, referred to as infant sibling). Comparing infant siblings with children without a family history of autism, numerous early autism behavioral markers, such as gestures (Bradshaw et al., 2021; Gordon et al., 2015; West et al. 2020), fine and gross motor skills (Choi et al., 2018; Lebarton & Landa, 2019; Lebarton et al., 2016; Rowberry et al. 2015) as well as object exploration (Burrows & Bodfish, 2021; Northrup et al., 2017; Sparaci et al., 2018), have been observed (see also Dawson et al., 2023 for a review). Overall, these behavioral prospective studies are based either on clinical reports (developmental, adaptive behavior scales, parent-report behavioral checklists) (Lee et al., 2019; Sacrey et al., 2020; Wetherby et al., 2021) or behavioral observations during semi-structured interactions, with an adult examiner providing specific probes to elicit a target behavior (Heymann et al., 2018; Zwaigenbaum et al., 2021​). Nevertheless, while these study designs seek to be rigorous and consistent, they may introduce biases related to assessments, recall bias, or may lack ecological validity, thereby overlooking the observation of spontaneous child behaviors in naturalistic settings such as during play with parents.

Notably, repeated evidence from early typical development tells us that naturalistic observational paradigms of social communication behaviors (e.g., vocalizations, gestures, eye-contact) are maximized when infants engage in familiar play interactions with the caregivers (Iverson et al., 1994; Thal and Tobias, 1992). While many researchers have argued for studying communication and social behaviors in young autistic children during natural and spontaneous interactions (e.g., Tager-Flusberg et al., 2009; Wetherby, 1986), surprisingly few studies have used an ecological paradigm of naturalistic caregiver-child play interaction, applying either a macro-analytical (i.e., global rating to assess general constructs) or a micro-analytical coding approach. The latter, also referred to as a ‘granular’ system (Delehanty & Wetherby, 2021), allows researchers to characterize target behaviors with respect to frequency, duration, and timing on a second-by-second basis (Morawska et al., 2015), enabling a deep understanding of the socio-communicative actions that characterize parent-child interactions. Research employing a micro-analytic coding methodology during natural caregiver-child interactions has identified several behavioral characteristics in infant siblings later diagnosed with autism. These features reflect distinctive patterns of early communication and interaction in autistic children, such as: reduced frequency of shared positive affect and conventional communicative gestures (Campbell et al., 2015; LeBarton & Iverson, 2016); a different approach to object exploration and manipulation (Mulligan & White, 2012); variations in how children coordinate communicative signals—such as gestures, vocalizations, eye gaze, and facial expressions (Parladé et al., 2015); differences in how gestures and speech are integrated during early communication (Choi et al., 2020). However, within the subset of studies utilizing a micro-analytic naturalistic approach to caregiver-child interaction, only a limited number have focused on toddlers already diagnosed with autism. These studies have highlighted differences in early communicative development among autistic children, including reduced production of conversational-interactive gestures (Mastrogiuseppe et al., 2015; Mishra et al., 2021), distinct patterns of engaging in joint attention (Papoulidi et al., 2021), and a reduced tendency to combine gestures with sounds or words relative to neurotypical toddlers (Delehanty & Wetherby, 2022). These findings point to diverse pathways of social communication, which may reflect unique ways of interacting with others and processing the environment.

More recently, research has sought to integrate computational and behavioral science in the field of autism. Machine learning (ML) techniques have been employed in these efforts to develop predictive models aimed at improving the timing and accuracy of an autism diagnosis (Akter et al., 2019; Tartarisco et al., 2021; Levy et al., 2017). These models have also been applied to uncover potentially distinct autism subgroups and to investigate response to interventions (Stevens et al., 2019; Usta et al., 2019). However, to the best of the authors’ knowledge, ML approaches have not yet been used to predict the accuracy of behavioral traits associated with autism, involving a micro-analytic and multi-domain approach derived from video recordings of naturalistic interactions.

Based on the present literature, our aim was to delve deeper into early behavioral characteristics that may distinguish autistic children from neurotypical children, within a comprehensive developmental framework. To achieve this, we needed first to unravel the complexity of behavior by shifting away from a single-domain approach and instead exploring the optimal weighting of combinations of behavioral features with a more comprehensive and nuanced perspective of behavioral dynamics. Hence, we conducted a detailed, fine-graded investigation of the very early potential behavioral cues of autism in a developmental and multidimensional framework and we applied ML techniques to evaluate whether and how multiple behavioral characteristics contribute to predict an autistic condition. Specifically, we rigorously coded, using a novel and detailed micro-analytic coding scheme, early motor actions, gestures, and speech in toddlers and young children with and without autism, both quantitatively (e.g., frequency or duration) and qualitatively (e.g., coordination of different behaviors such as gesture-gaze integration), during naturalistic parent-child interactions.

We hypothesize that certain behavioral cues, when considered in combination, will exhibit distinctive patterns that correlate with autistic neurodivergent or neurotypical developmental profiles. We further anticipate that integrating the microanalytic approachwith ML would enhance our ability to detect behavioral patterns that characterize both autistic and neurotypical developmental profiles.

## Methods

### Participants

A total sample of 58 toddlers and young children, 30 autistic (ASC) and 28 neurotypical (NT) children (12 females in each group), participated in the present study. ASC and NT children were individually matched according to the ASC child’s non-verbal developmental age (ASC: mean= 22 months, sd=8.3; NT: mean= 20 months, sd= 5.7) as assessed by the Performance subscale of the Griffiths Mental Development Scales – Third Edition (GMDS-III). All children were exposed primarily to Italian at home. ASC children and their families were recruited through and participated in naturalistic caregiver-child interactions at the National Research Council of Italy, Institute for Biomedical Research and Innovation (CNR-IRIB) in Messina and at the clinical and territorial service in Catania. NT children were recruited via mainstream nursery schools in the local territory of Messina and Catania. All autistic children had received a diagnosis of Autism Spectrum Disorder according to DSM-5 criteria within a multidisciplinary team and supported by the Autism Diagnostic Observation Schedule – Second Edition (ADOS-2; Lord et al., 2012). For children assessed before 30 months of age, diagnostic assessments were re-evaluated longitudinally within the clinical follow-up program, and only children whose autism diagnosis remained stable over time were included in the study.

The Griffith’s Mental Development Scale- III (GMDS; Green et al., 2016) was administered to assess the different domains and overall developmental level. Adaptive functioning in daily life was evaluated using the Vineland Adaptive Behavior Scales, Second Edition (VABS-II; Sparrow et al., 2005), which covers four domains: communication, daily living skills, motor skills, and socialization. Language development was assessed using the Primo Vocabolario del Bambino (PVB; Caselli et al., 2015), the Italian adaptation of the MacArthur-Bates Communicative Development Inventories, Second Edition (MB-CDI; Fenson, 2006). The PVB collects information on early communicative and linguistic development, starting with initial gestural signals and progressing through vocabulary expansion, the emergence of grammar, and the first word combinations.

Inclusion criteria for the ASC group required a primary diagnosis of autism and the absence of medical or neurological conditions known to independently affect neurodevelopment. Exclusion criteria included: known genetic syndromes associated with neurodevelopmental impairment, early-onset epileptic encephalopathy, significant sensory or motor impairments, severe prematurity or low birth weight, and clinical profiles meeting criteria for primary non-autistic neurodevelopmental disorders, such as global developmental delay (GDD) or developmental language disorder (DLD). Given the heterogeneity of early autism, the presence of co-occurring developmental vulnerabilities frequently associated with autism (e.g., language delay, uneven cognitive profiles, emerging attentional regulation difficulties) was systematically evaluated but not treated as exclusionary, provided that autism was clearly identifiable as the primary neurodevelopmental condition. To support this distinction, all ASC children underwent standardized assessments across cognitive (GMDS-III), adaptive (Vineland Adaptive Behavior Scales – Second Edition, VABS-II), and communicative (Primo Vocabolario del Bambino, PVB) domains. These measures were used to characterize individual developmental profiles and to exclude children whose functioning patterns were more consistent with alternative primary neurodevelopmental disorders.

NT children were recruited from mainstream nursery schools in the same geographical area. Inclusion criteria required typical developmental milestones and age-appropriate adaptive, social, and communicative functioning. NT children had no history or clinical suspicion of neurodevelopmental disorders, including autism, DLD, GDD, or attention-deficit/hyperactivity disorder. Typical development was confirmed through standardized parent-report measures of adaptive functioning (VABS-II) and language development (PVB). Only children whose scores fell within the expected normative range for age were included in the NT group.

Demographic and clinical characteristics of the sample are reported in Table 1.

**Table 1:** Demographic and clinical information of the ASC and NT groups.

	*ASC* *(N = 30)*	*NT* *(N = 28)*	*U*	*p-value*
***Demographics***				
Sex (M/F)	18/12	16/12	-	0.825
Age (months)	26.3 (5.2)	20.3 (5.7)	177	<0.0001****
GMDS-ER Performance AE	21.5 (8.3)	-	-	-
***PVB***^a^				
Exp-LQ	63 (14.2)	98 (30.4)	63	<0.0001****
Rec-LQ	87 (17.8)	116 (35.1)	72	<0.0001****
AGQ	68 (18.6)	95 (23.8)	61	<0.0001****
***VABS-II standard score***^b^				
ABC	69 (11.7)	101 (21.6)	11	<0.0001****
Communication	62.2 (11.2)	96.4 (21.9)	10.5	<0.0001****
Daily living skills	73.2 (10.5)	101.9 (20.7)	14	<0.0001****
Socialization	66.5 (10.5)	98.7 (20.7)	2.5	<0.0001****
Motor	91.9 (12.4)	101.9 (19.9)	135	0.0001***
***ADOS-2***^c^				
SA	13.8 (3.8)	-	-	-
RRB	2 (1.7)	-	-	-
CSS	5.6 (1.55)	-	-	-
***GMDS-ER DQ score***^d^				
General	71 (11.8)	-	-	-
Hearing and Language	47 (5.7)	-	-	-
Personal Social	70 (13.9)	-	-	-
Performance	81 (23.4)	-	-	-
Eye-hand Coordination	72 (11.7)	-	-	-
Locomotor	86 (16)	-	-	-

Values for age and standardized measures report mean (SD) values.

Abbreviations: PVB, Primo Vocabolario del Bambino; Exp-LQ, expressive vocabulary quotient; Rec-LQ, receptive vocabulary quotient; AGQ, Actions and Gestures Quotient; VABS-II, Vineland Adaptive Behavior Scales II Ed.; ABC, Adaptive Behavior Composite; ADOS-2, Autism Diagnostic Observation Schedule-2; SA, Social Affect, RRB, Restricted Repetitive Behaviors, CSS, Calibrated Symptom Severity score; GMDS-ER, Griffiths Mental Development Scales-Extended Revised; DQ, Developmental Quotient; AE, Age Equivalent.

a number of subjects ADOS-2 = 17

b number of subjects GMDS = 17

c number of subjects PVB (ASC=10; NT= 15)

d number of subjects VABS-II (ASC=11; NT= 15); Signif. codes: 0 ‘***’ 0.001 ‘**’ 0.01 ‘*’

The study received ethical clearance by the local health ethics committee (protocol number 08/2021), and all the caregivers provided informed consent to participate in the study.

## Procedure

### Parent-child interaction protocol

**Fig. 1:**
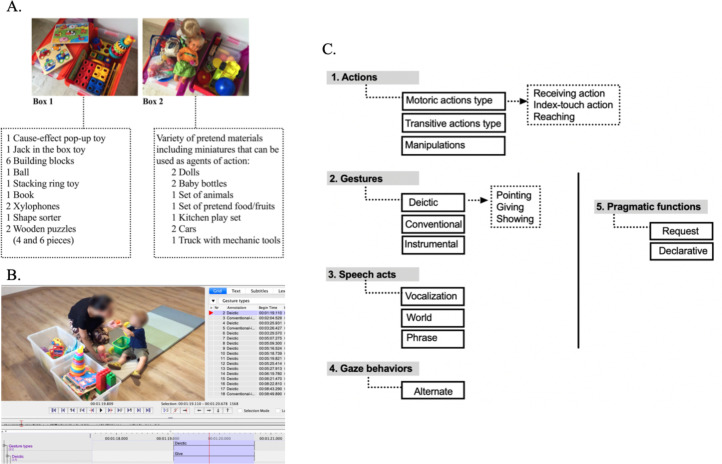
A. List of parent-child interaction toys and materials. B. Screenshot of the ELAN annotation system (written consent to use the image has been obtained). C. Microanalytical coding scheme hierarchical architecture.

All mothers and children participated in a Parent-Child Interaction (PCI) protocol conducted before they started a Naturalistic Developmental Behavioral Intervention (NDBI) following the principles of the Early Start Denver Model (ESDM). The PCI protocol consists of a 10-minute free play interaction, during which mothers are given a standardized set of age-appropriate toys and instructed to engage with their child as they would at home. The PCI toy set is organized into two boxes providing a diverse array of play materials including duplicates to promote reciprocal play (Fig. 1A). The PCI interactions took place in a quiet room without any other play material or distractions. A square carpet approximately 2 × 2 m was placed on the floor to allow the participants to sit naturally and comfortably. The two boxes, without lids, were placed on one side of the carpet so as to be easily accessible by both child and parent. The interactions were recorded through a high-resolution digital camera, which was operated by the researcher. The researcher maintained an appropriate distance to ensure a frontal perspective of the scene. Under no circumstances did the researcher engage with either the parent or the child to offer information or influence the interaction. Special care was taken to ensure that the researcher’s presence did not interrupt the natural flow of the interaction.

### Behavioral micro-analytic coding

Video footage of all the mother-child interactions was carefully reviewed to be transcribed for detailed analysis of actions and multimodal communication. Relevant behavioral information from video contents were extracted using an open-source behavioral annotation tool for audio and video recordings, which allows an unlimited number of textual annotations, supports creation of multiple tiers and tier hierarchies (Eudico Linguistic Annotator, ELAN; Sloetjes and Wittenburg, 2008). See Fig. 1B for an overview of the ELAN annotation system.

A novel micro-analytical hierarchical coding scheme, specifically designed for the study, was implemented. It examined various motor schemes and communication modalities for social orienting, social responsiveness, and social initiative. The analysis included the frequency and/or duration of target behaviors, such as motor actions, gestures, and speech. Subcategories of these behaviors, such as reaching behavior within motoric actions or pointing, giving, showing, conventional-interactive and instrumental within gestures, were also explored. Additionally, the study investigated chained behaviors, such as object-partner alternate gaze associated with gesture, along with the pragmatic functions of gestures (i.e. request of objects or help, versus declarative). Fig. 1C presents a comprehensive depiction of the coding scheme.

In the Supplementary Materials, Section S1 provides detailed information on the coding manual, including descriptions of the behavioral characteristics, while Table S1 presents the descriptive statistics of the behavioral variables for the ASC and NT groups.

Four coders analyzed the videos. Before beginning the video analysis, inter-coder reliability was established on 20% of the videos (Cohen’s kappa values: .72 for actions, .80 for gestures, .71 for speech; Landis and Koch, 1977). Specific information on the coding training procedures and the assessment of inter-coder reliability is provided in Section S2.1 of the Supplementary Materials.

### Data analysis overview

All statistical analyses and visualizations were conducted using R (v. 4.2.1). As a first step, we performed a Principal Component Analysis (PCA) to reduce the dimensionality of the dataset and to visually explore the most informative variables. A biplot of the first two components (PC1 and PC2) was used to visualize group distribution and explore variable contributions.

Subsequently, to select features for classification, we applied a multi-step procedure performed independently within each fold of the cross-validation thereby avoiding information leakage: highly correlated variables (r > 0.70) were screened using Pearson and point-biserial correlations to reduce redundancy and retain those most predictive of group membership. Frequency-based variables were log-transformed and all features were standardized. An ANOVA F-test was then used to retain only variables with significant group differences (*p* < 0.05). Using the selected features, we trained a logistic regression (LR) classifier with both 10-fold and leave-one-out cross-validation. Classification performance was evaluated using Accuracy, Precision, Recall, and F1 Score. SHAP values were computed to assess feature importance and interpret model predictions. A full description of the procedures, feature selection steps, and model interpretability analyses is available in the Supplementary Materials (S2.2).

Finally, as a secondary analysis, we conducted correlation analyses to examine the relationships between the behavioral variables that primarily overlapped between NT and ASC participants in the third quadrant of the PCA and chronological age in both groups, as well as performance mental age in the ASC group.

## Results

Upon closer examination of the variables’ correlation with the principal components, and observing sample projections in the new space, a distinctive pattern became apparent. Specifically, NT children predominantly occupy quadrants characterized by positive correlations with: (a) greater gesture use: this includes not only a higher frequency of pointing, showing, giving, and conversational-interactive gestures but also multimodal integration, such as gesture-gaze coordination; (b) higher verbal communication: NT children in these quadrants exhibit more spoken words and multiword utterances (c) increased engagement in functional play schemes: this involves a greater involvement in functional play schemes with objects.

**Fig. 2:**
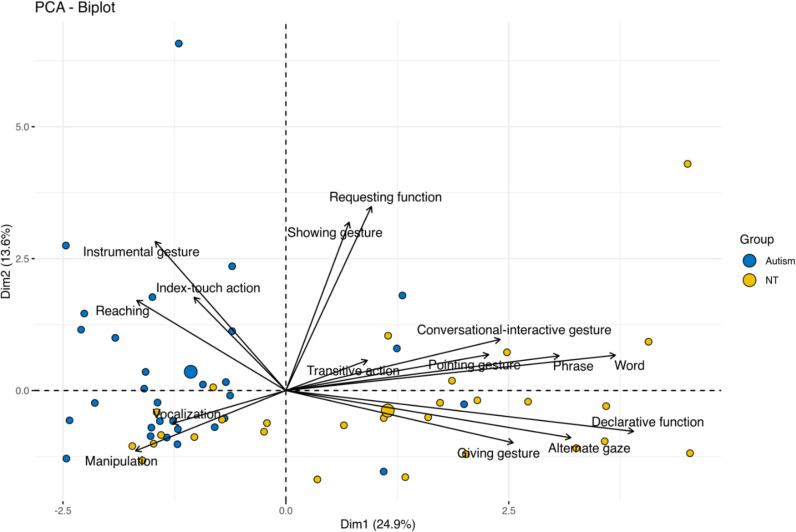
Two-dimensional scatter plot of ASC and NT children’s behavioral features, projected onto PC1 and PC2 plane.

Conversely, ASC children predominantly exhibited a positive correlation with variables indicating: (a) a distinctive gestural profile, characterized by fewer conversational-interactive and showing, giving, or pointing gestures, and a greater use of instrumental gestures; (b) presence of motor actions, including reaching behaviors, hand opening to receive an object from the partner, as well as the use of the index finger mainly to carry out motor actions on objects; (c) lower frequency of partner-object alternate gaze during gesturing; (d) greater use of vocalizations, rather than words or phrases; (e) higher object manipulation, with less engagement in transitive functional actions. Together, these patterns highlight distinct behavioral profiles that differentiate ASC and NT groups. Fig. 2 shows the behavioral characteristics of the ASC and NT children projected onto the PC1 and PC2 plane.

The final subset of features (see Supplementary Materials, S2.2) that, after the grid search, most frequently emerged as the best combination of predictors of autism condition included ‘Alternate Gaze’, ‘Reaching’, and ‘Instrumental Gesture’. Although slight variations in feature selection occurred across the LOOCV folds, these three variables were consistently among the most selected. Specifically, in the LOOCV setting, ‘Reaching’ was included in 100 % of folds, while ‘Instrumental Gesture’ and ‘Alternate Gaze’ appeared in approximately 95% of them. Similarly, in the 10-Fold Cross-Validation, ‘Reaching’ was always selected, with ‘Instrumental Gesture’ and ‘Alternate Gaze’ being retained in 80–90% of the folds.

Using these selected features as input for the Logistic Regression (LR) model aimed at classifying ASC and NT children, we attained an accuracy of approximately 80%. A comprehensive overview of the model’s performance metrics for both Leave-One-Out and 10-Fold Cross-Validation is provided in Table 2.

**Table 2:** Summary of model performance: leave-one-out and 10-fold cross-validation.

	Cross-Validation Method	
Measure	Leave-one-out (average)	10-folds (average, SD)
Accuracy	79.31%	84.67±9.09%
Precision	82.14%	92.50±11.46%
Recall	76.67%	80±22.11%
F1 Score	79.31%	82.71±13.13%

**Fig. 3:**
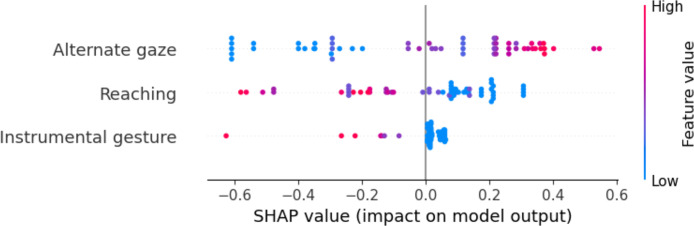
Beeswarm plot displaying SHAP values for the top four features.

SHAP values associated with the key features were consistent with the PCA-derived patterns. As depicted in the beeswarm plot presented in Fig. 3, elevated values for the features ‘Alternate Gaze’ exert a positive influence on the model output, leaning it towards the NT group. Conversely, for the remaining two features, namely ‘Reaching’ and ‘Instrumental Gesture’, higher values guide the model toward the classification of ASC children. This analysis clarifies how each feature contributes to the model’s discriminative decisions.

As secondary analyses, the PCA biplot revealed that NT and ASC participants showed partial overlap in the third quadrant, primarily influenced by Vocalization and Manipulation. To further examine this pattern, Spearman correlations with Chronological Age (CA) were computed within each group. In the NT group, Manipulation was negatively correlated with CA (ρ = –0.532, *p* = 0.004), while no significant effects emerged for Vocalization. In the ASC group, neither CA nor Performance Mental Age was significantly associated with Vocalization or Manipulation. These results suggest that the observed group differences are not solely attributable to chronological age or general developmental level.

## Discussion

This study adopted a comprehensive framework to characterize early autism by examining the contribution of multiple behavioral features. It systematically explored a wide range of behaviors, including manipulation, transitive actions, gaze, gesture, and speech, along with their multimodal integration, within an ecological paradigm of naturalistic parent-child play interactions in very young neurodivergent autistic and neurotypical children. Using an innovative micro-analytic approach, diverse behavioral signatures were extracted. Machine learning techniques were applied to pinpoint optimal combinations of variables, characterizing individual-level behavioral profiles. These multidimensional markers were subsequently employed to identify patterns within the sample, resulting in distinct clusters differentiating autistic and neurotypical children.

The findings underscore qualitatively different developmental profiles, reflected in variations in the use of actions, gestures, and verbal communication during interactive play. Such differences highlight the diverse modalities through which children engage socially and explore their environment.

Specifically, the NT cluster predominantly displayed a broader and more frequent use of gestures, greater modulation of gestures with eye contact, a higher number of spoken words and phrases, and increased engagement in functional play with objects. In contrast, the ASC cluster revealed a distinct and meaningful developmental profile, marked by alternative communicative strategies—such as a greater reliance on gestures and actions with a requesting or regulatory function (i.e. reaching behaviours and instrumental gestures), emerging motor precursors to pointing, and less frequent use of pointing. Gesture use was less modulated by gaze, and communication often involved more vocalizations than words. Play was characterized by an increased focus on object manipulation, reflecting a different way of engaging with and exploring objects, rather than conventional functional play schemes.

Significantly, we identified three pivotal behavioral features alternate gaze, reaching and instrumental gesture that hold the utmost predictive power for the automated identification of children with and without autism. Applying these markers, the classifier achieved remarkable discrimination between the two groups, with accuracy and precision rates exceeding 80%. Notably, istrumental gestures, in which the child physically guides another person’s hand or arm to prompt a specific action, were observed exclusively in autistic children and not in NT peers, highlighting their potential relevance as a distinctive behavioral characteristic within this sample. Unlike more socially oriented gestures such as pointing or conversational-interactive gestures which were significantly reduced in autistic participants, instrumental gestures reflect a goal-directed strategy for regulating another person’s actions, providing insight into alternative interactive modes.

Their uniqueness and rarity in the existing literature make them particularly noteworthy: to date, only two other studies (Mastrogiuseppe et al., 2015; Mishra et al., 2021) have reported similar findings in a naturalistic interaction context. The presence of such gestures, especially when coupled with the reduced use of other communicative forms, may point to a qualitatively different developmental trajectory in early social interaction in autism. Additionally, reaching behaviors—described in the literature as potential precursors to communicative gestures with deictic qualities (Liszkowski & Ruether, 2021)—were more frequently observed in the ASC group, reflecting an alternative pathway of expressing needs and intentions, particularly in relation to object-directed communication. Rather than indicating immaturity, these behaviors may represent a different orientation toward interaction, more focused on goal-directed engagement. NT children, by contrast, tended to display gesture patterns such as deictic and conventional-interactive gestures accompanied by alternating gaze toward the caregiver.

Our correlation analyses provide additional insight into the role of chronological age in shaping early behavioral patterns. In NT children, fine-motor exploration followed the expected developmental trend, with Manipulation decreasing as age increased. In contrast, the lack of age-related associations in the ASC group suggests that these behaviors may be less closely linked to maturational processes in early development. The absence of correlations with age for Vocalization in both groups also indicates substantial interindividual variability and limited age dependency within the developmental period examined. Overall, these findings suggest that age alone does not fully account for the observed group differences, supporting the possibility of distinct developmental organizations in early communicative behaviors in ASC and NT children.

These behaviors are typically interpreted as signaling a desire to share experiences and initiate social interaction, highlighting different but equally meaningful approaches to early communication. While our study aligns with existing evidence highlighting early indicators of autism across various developmental domains, including actions, gestures, and speech, it emphasizes the need to move beyond the prevalent single-domain approach evident in previous research (see Leezenbaum et al., 2014; Mulligan & White, 2012; Paul et al., 2011). Only a limited number of studies have examined coordination of multiple communicative behaviors, such as gestures, vocalizations, gaze, and smiles (as explored in Parladè et al., 2015) or gesture-speech integration (see Choi et al., 2020) during naturalistic parent-child interactions with infants with an Elevated Likelihood of developing autism (EL). Moreover, while prospective studies on EL infants have provided valuable insights into the earliest stages of life, it remains unclear whether findings from this subgroup can be generalized to the broader population of autistic young children (Dawson et al., 2023). Hence, this study fills a gap in the literature by advancing prior research through a combined micro-analytical and multi-domain approach, while using machine learning algorithms to identify the most influential subset of behavioral features capable of robustly predicting an autism condition.

Despite its time-intensive nature, this approach offers unique and detailed insights into early actions and multimodal communication in autism within a naturalistic, developmental, comprehensive framework. Furthermore, by leveraging advanced data science methods, we were able to identify optimal weightings for combinations of behavioral variables. This contributes to a nuanced understanding of how multiple markers effectively characterize autism at an early developmental stage and which is the best subset of variables that can efficiently discriminate between young ASC and NT children.

The study exhibits certain limitations that necessitate careful consideration and highlight areas for future research. Firstly, the absence of a comparative analysis with other clinical groups highlights the need for broader exploration to better understand whether the observed developmental features are specific to autism or shared across different neurodevelopmental profiles. Secondly, limited sample size of ASC children hinders exploration of potential subtypes within the autism spectrum. A further limitation concerns the absence of direct matching on demographic variables such as socioeconomic status (SES). Although SES was not measured and residual confounding cannot be fully ruled out, all participants were recruited through the same clinical and community services within a single geographical area, which reduces the likelihood of substantial SES-related variability across groups. The two groups were intentionally aligned on non-verbal developmental level and sex, consistent with the study’s aim of comparing behavioral organization at comparable functional stages; however, the lack of explicit SES control remains a constraint and should be addressed in future research. Addressing these limitations and paving the way for future research, our study offers an opportunity to delve into both quantitative and qualitative behavioral dimensions characterizing early autism. In particular, given the high prevalence of instrumental gestures observed in our autistic group, future research might investigate in detail the nature of these communicative behaviors and their significance. Furthermore, considering the heightened occurrence of reaching behavior in our sample, acknowledged as a precursor to deictic request gestures, forthcoming research could undertake a longitudinal investigation of individual behavioral trajectories involving early precursors, communicative gestures, and speech. This could provide valuable insights into the developmental pathways and relationships among these key aspects, enhancing our understanding of early autism. Last but not least, future research in this field may involve the development of artificial intelligence models designed to automatically identify and quantify these behavioral characteristics from naturalistic videos, as done in a recent pilot study for the recognition of deictic gestures from naturalistic videos using a transformer-based model (Bruschetta et al., 2023).

The present findings hold important implications not only for early identification of autism but also for the development of supportive, strengths-based approaches to early intervention. The behavioral markers identified—particularly differences in gesture use, multimodal coordination, and fine-motor exploration—offer meaningful insight into early communicative processes and highlight domains that may be especially responsive to parent-mediated strategies. Observing reduced gesture production, for instance, can guide clinicians in helping caregivers create interactional contexts that expand opportunities for coordinated communication. Likewise, the patterns emerging from our analyses suggest the value of interventions that scaffold exploratory actions and gradually support the transition toward more intentional communicative forms. Integrating these early behavioral signatures into tailored support plans may foster more attuned caregiver–child exchanges, promote engagement through developmentally sensitive, play-based interactions, and ultimately contribute to more adaptive early developmental pathways that respect each child’s individual strengths and modes of expression.

## Conclusions

Diverging from single-domain approaches, our study innovatively introduced a holistic analysis encompassing various motor and socio-communicative behaviors to enhance the early characterization of autism. Utilizing an innovative micro-analytic approach during naturalistic parent-child play interactions, our findings revealed distinct profiles between autistic and NT children. In particular, ASC children displayed reduced production of deictic and conversational–interactive gestures, increased use of instrumental gestures, more frequent reaching behaviors and index-finger object actions, greater object manipulations, more vocalizations over words, and reduced gaze alternation between the social partner and objects while gesturing.

Moreover, identifying the most discriminative behavioral features for automated identification in both autism and NT groups, we found that alternate gaze most strongly characterized NT children, while instrumental gestures and reaching more consistently characterized autistic children. By adopting an overarching developmental framework, our study offers a comprehensive understanding of the diverse behavioral expressions associated with autism, contributing to a more attuned recognition of individual profiles and to the development of supportive, personalized approaches.

## Author contributions

Conceptualization: LR, MM, OC, GT, GP. Data curation: RB, MM, EL, AC. Methodology: LR, MM, GT, OC. Formal analysis: MM, RB, SC, GT. Investigation: FIF, EL, SA, AC, CC, OC, LT, GDR, The NEST Team. Supervision: LR, MM, OC. Writing - original draft preparation: MM, FF, RB. Writing - review and editing: LR, MM, GT, OC, MVL, GP. Visualization: RB, SC, GT. Funding acquisition: LR, GP. Project administration: LR, GP.

## Ethical considerations

The study received ethical clearance by the local health ethics committee (protocol number 08/2021), and all the caregivers provided informed consent to participate in the study.

## Funding statement

This project was supported by the INTER PARES project, EU POC METRO 2014/2020 (Azione l.3.1. – Codice Progetto ME I.3.1.b.); EARLY START project (Deliberazione n.1508/2021); READS project (MIMIT, Prog. n. F/180026/01-04/X43); DREAM project (PRIN – MUR, DSB.AD008.649); SAMOTHRACE project - Sicilian Micronano Tech Research and Innovation Center - Ecosistema dell’Innovazione - ECS00000022 - CUP B63C22000620005; MM is partially supported by HEALING project (PRIN- MUR, P2022SMEJW).

## Data availability statement

The data that support the findings of this study are available from the corresponding author upon request.

## Ethics approval and patient consent statement

The study received ethical clearance by the local health ethics committee (protocol number 08/2021), and all the caregivers provided informed consent to participate in the study.

## Declaration of competing interest

The authors declare that there are no conflicts of interest

## Appendix Supplementary materials

Image, application 1
