# Supplementary material for: Early multimodal behavioral cues in autism: a micro-analytical exploration of actions, gestures and speech during naturalistic parent-child interactions
Source: Int J Clin Health Psychol. 2026 Jan 22;26(1):100664. doi: 10.1016/j.ijchp.2026.100664 (PMC12861202; doi:10.1016/j.ijchp.2026.100664)
Supplement: Supplementary file 1 [file mmc1.docx]

**Supplementary Information**

**S1 - Behavioral coding scheme: variables description**

All behaviors exhibited by the children were categorized into one of the following categories:

1.**Manipulation:** Generic manipulations and explorations of the object encompass activities involving a non-specific motor scheme, such as mouthing, rotating, shaking, banging, or turning an object. This category also includes tactile exploration, like touching or scraping to examine surface characteristics, particularly when objects have grooves or diverse textures. Visual-only exploration is considered only in explicit cases where the child picks up an object and deliberately moves their head to view it from different angles.

2.**Actions**

Were classified into one of the following categories:

**Motor actions:** include the following subcategories:

***Index-touch action:*** This category involves motor patterns requiring the use of the index finger to interact with an object. Examples include pressing a pop-up with the index finger, touching a doll's foot with the index finger for exploration, or popping soap bubbles with the index finger.

***Reaching***: This behavior is characterized by an attempt to stretch or extend their arm toward an object of interest, held by the partner, with associated trunk, arm, and hand tension directed forward. Following Thelen et al. (1993), an arm movement will be coded as a reach if: a) an object is located in the infant's reachable space; b) the infant looks at the object before reaching; and c) one or two hands contact the object (i.e. successful grasp is not required).

***Receiving action***: The child accepts an object that the adult is explicitly offering.

**Transitive actions:** This category encompasses all actions with objects, including using a concrete object appropriately (e.g., bringing a miniature cup to the mouth, stacking circles) and inventive use, where the child substitutes one object for another (e.g., bringing a wooden cube to the mouth, using a fork as a comb).

3.**Gestures**

Were classified into one of the following categories:

**Showing:** A gesture is defined as showing when one holds up an object in the partner’s line of sight.

**Giving:** A gesture is defined as giving when one gives an object to their partner.

**Pointing:** A gesture is defined as pointing when there is a clear extension of the arm, hand, and index finger directed toward a specific object, location, or event.

**Conventional-interactive:** Are arbitrarily related to their meanings, culturally defined, and used for the purpose of regulating interaction (e.g. nodding one’s head for “yes,” shaking one’s head for “no,” shaking one’s extended hand for “more” or “less,” or moving both hands back and forth with one’s palms toward a partner for “wait”).

**Instrumental:** These gestures are used to involve the partner in the action, utilizing the partner's body as a tool to achieve an immediate goal, aiming to prompt the partner to take immediate action (e.g. the child takes their or her mother’s hand and brings it closer to the desired object, or the child takes their mother’s hand and places it on the door to indicate the desire to go out).

4.**Pragmatic functions**

Each gesture produced by the child has been systematically coded to align with its corresponding pragmatic function.

**Requesting function**: Is assigned when the child employs a gesture to make a specific request, such as pointing to an object to indicate a desire for their mother to give it to them.

**Declarative function**: Is assigned when the purpose of the gesture is to express shared interest in an object or event with someone, such as the child pointing to an object to convey their enthusiasm to an adult.

5.**Alternate gaze**

Child’s gaze behavior is specifically analyzed when it is accompanied by a gesture. The classification of gaze behavior as "alternate" occurs in two scenarios: firstly, when the child's gaze alternates between the focus object (i.e., the object referred to by the gesture), the partner's face, and the focus object itself, or vice versa); secondly, when the child's gaze shifts from the focus object to the partner's face, or vice versa.

**6.Speech acts**

Vowel utterances were classified into the following three main categories: vocalizations, words, and phrases:

**Vocalizations**: Vocalizations encompassed vowel strings (e.g., “eeaa”), reduplicated babble (e.g., “gaga”), and variegated babble (e.g., “bama”), while non-vowel sounds like laughter, crying, and vegetative noises (e.g. sneezing, coughing, breathing) are not coded.

**Words**: Include verbal productions referring to a specific referent on multiple occasions or in different contexts, phonetically similar to the adult model. This category encompassesd actual Italian words (e.g., "ball," "cow," "yes," "no"), consistent sound patterns used by a child for a specific object or event (e.g., using "pa" to refer to a ball), articles, onomatopoeic sounds systematically used for specific referents (e.g., "woof," "meow," "choo choo"), and evaluative sounds (e.g., "wau!", "hej!", "oh-oh!").

**Phrases**: Phrases consist of the sequential combination of two or more individual intelligible words in verbal productions.

Table 1 presents the descriptive statistics for the behavioral variables outlined above, reported separately for the ASC and NT groups. Values are reported as counts for all variables, except for Manipulation and Transitive Action, which are reported as durations in milliseconds.

***Table S1. Descriptive statistics of behavioral variables for the ASC and NT groups.***


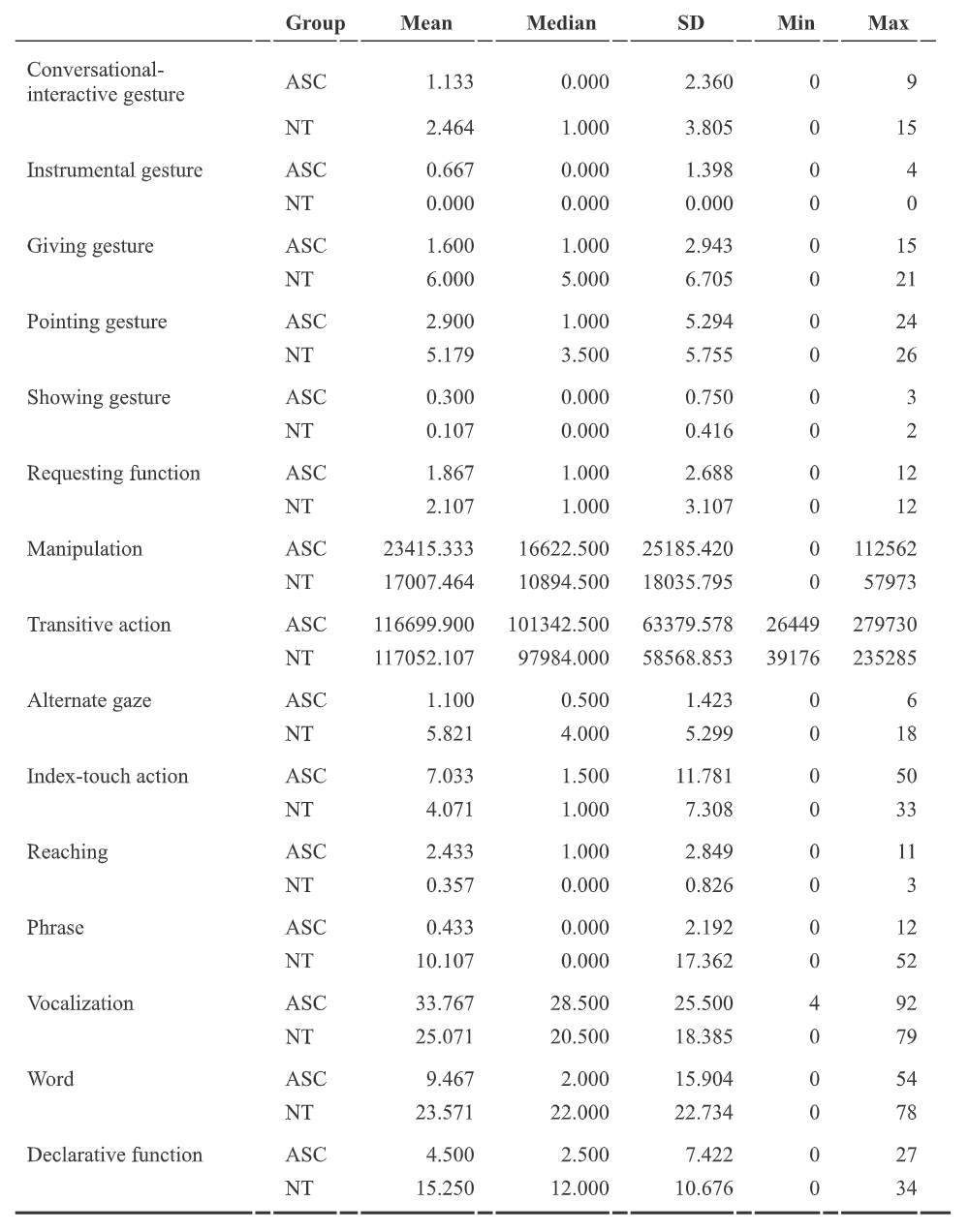


**S2. Data Analysis**

***S2.1 Inter-coder reliability***

During an initial training phase, four coders underwent formal training, supervised by an expert in the coding procedure. Organized sessions were conducted to familiarize coders with the ELAN software, offering guidance on its usage and facilitating the exploration of selected target behaviors. This involved coding four videos featuring both NT and ASC children. After the training phase, each coder was tasked with independently coding twelve randomly selected video clips (6 ASC, 6 NT), constituting 20% of the total videos. The coding was conducted in a blinded manner to the autism condition. The expert independently coded the same video segments and computed Cohen’s Kappa (Cohen, 1960) as a reliability metric. Cohen’s Kappa was determined through pairwise comparisons between the expert and each independent coder individually. Subsequently, an average Kappa was calculated for each of the macro categories considered in the analysis. Cohen’s kappa values were .72 for actions, .80 for gestures, .71 for speech, reaching a level considered "substantial" (Landis and Koch, 1977). Disagreement locations were pinpointed and collaboratively reviewed and resolved by both coders and the expert. To ensure intercoder reliability, weekly meetings were held among the experts and coders. These meetings aimed to sustain agreement on macro categories, establish consensus on hierarchical categories, validate the coding process, and address any uncertainties or concerns about the coding procedure.

***S2.2 Procedures, feature selection steps, and model interpretability analyses***

All statistical analyses and graphical visualization were implemented in R (version 4.2.1). To comprehensively explore multiple target behaviors, we conducted a Principal Component Analysis (PCA) as a first step. The aim was to extract the most relevant information from our rich data set by efficiently reducing the number of variables. This reduction facilitated clearer visualization and interpretation of the data, revealing its inherent patterns. Specifically, we projected the data onto a condensed set of principal components to explore their effectiveness in discriminating between ASC and NT children. A scatterplot was created for the participants using the first two principal components (PC1 and PC2) and different colors were assigned to children in each group. Additionally, to evaluate the influence of each original variable on the first two principal components, we overlaid a correlation plot of the variables onto PC1 and PC2. Variables that have stronger correlations with PC1 and PC2 have greater relevance in explaining variability within the dataset. Consequently, these variables were utilized as input to train the Logistic Regression (LR) classifier for automated differentiation between the ASC and NT groups. LR was selected as the primary classifier because it provides an appropriate balance between interpretability and predictive performance in modest sample sizes, while reducing the risk of overfitting typically associated with more complex non-linear models. Alternative classifiers (e.g., Random Forests, non-linear SVMs, gradient-boosting algorithms) were considered but were deemed less suitable given the dimensionality of the feature space and the study’s emphasis on model transparency. To maintain parsimony, interaction terms and non-linear transformations were not included; however, the feature-selection procedure was designed to identify the most informative additive contributions across cross-validation folds. SHAP analyses further supported interpretability by illustrating how selected features jointly influenced classification outcomes despite the linear nature of LR. Together, LR and SHAP allowed us to capture coordinated patterns among behavioral markers, consistent with the study’s objective of identifying meaningful multimodal signatures distinguishing ASC and NT developmental profiles.

Prior to model training, we applied a multi-step feature selection procedure designed to reduce redundancy, control multicollinearity, and preserve variables with the strongest association to the outcome. First, we computed pairwise Pearson correlations among all features and identified highly correlated pairs (r > 0.70). To avoid multicollinearity and retain the most informative variables, we calculated the point-biserial correlation between each feature and the binary outcome variable (ASC vs NT) and discarded the feature with the lower absolute correlation (Chan et al., 2022). This approach allowed us to prioritize features that are not only statistically independent, but also more relevant for group discrimination.

Next, we applied a log transformation (X’=log(1+X)) to all variables representing frequency or count data, in order to mitigate the effects of positive skewness and stabilize variance. This transformation is commonly used in behavioral data to improve distributional properties and prepare features for statistical testing (Feng et al., 2014). All features were then standardized (zero mean, unit variance) to ensure comparability and prevent scale-related biases during model training (de Amorin et al., 2023).

We subsequently employed an ANOVA F-test to rank the standardized features based on their discriminative power between the two groups (Elssied et al., 2014). This statistical test, suitable for continuous numerical variables and categorical group comparisons, identifies variables whose mean values differ significantly across classes. Only features with p-values below the conventional 0.05 threshold were retained for further analysis, ensuring that the selected subset included variables with statistically meaningful group-level differences. Among these, we conducted a grid search to identify the optimal combination of features that maximized classifier performance, thus avoiding arbitrary selection and overfitting.

Using the selected features as input, we proceeded to train and test the LR classifier using two different cross-validation methods: 10-fold cross-validation (10FCV) and leave-one-out cross-validation (LOOCV) to ensure that classification performance was consistent across different validation strategies.

No additional matching criteria (e.g. recruitment site) were applied at the preprocessing stage; however, to address the modest difference in group size between ASC and NT children, Logistic Regression was trained using class-balanced weighting to prevent bias toward the majority class. Furthermore, all cross-validation procedures were implemented in a stratified manner, ensuring proportional representation of both groups in each fold and reducing the risk of inflated or unstable accuracy estimates related to class imbalance. To report the outcome of the classifier, we applied the following performance metrics (Hossin et al., 2015), considering ASD as a positive class:

1. Accuracy: calculates the ratio of correct predictions to the total number of instances, providing an overall measure of predictive correctness.
2. Precision: is the ratio of true positive instances to the total instances classified as positive; a higher precision indicating a lower rate of false positives.
3. Recall: measuring the proportion of positive instances correctly classified, it signifies the classifier’s ability to identify all positive samples.
4. F1 Score: being the harmonic mean of precision and recall, provides a balanced measure where the contributions of precision and recall are equally considered.

To enhance the interpretability of our model results, we employed SHAP values (SHapley Additive exPlanations) (Lundberg et al., 2017). This game-theoretic approach provides valuable insights into how the essential features of a dataset influence the model’s output. In the beeswarm plot, each sample is represented as a dot, with its x-coordinate indicating the SHAP value for the respective feature. The color of each dot reflects the original value of the feature. Features are organized based on their predictive power, offering a visual representation of each feature’s impact on the model’s predictions.

Applying the feature selection algorithm on the behavioral variables to identify the most influential subset, we first excluded highly correlated features to avoid redundancy. Specifically, ‘Declarative Function’ was removed due to high correlation with ‘Alternate Gaze’, and ‘Word’ was discarded because of strong collinearity with ‘Phrase’. Among the features that emerged as statistically relevant based on p-values lower than 0.05 were, in ascending order of p-value: ‘Alternate Gaze’, ‘Reaching’, ‘Phrase’, ‘Giving Gesture’, ‘Instrumental Gesture’, and ‘Pointing Gesture’.
